# Eleven-Year Undiagnosed Bronchial Foreign Body in an Adult: A Case Report

**DOI:** 10.7759/cureus.115937

**Published:** 2026-09-08

**Authors:** Abdurrahman Jilani, Mohammed Mudassir Khan

**Affiliations:** 1 Medicine, Shadan Institute of Medical Sciences, Hyderabad, IND; 2 Medicine, Ayaan Institute of Medical Sciences, Hyderabad, IND

**Keywords:** airway foreign body, delayed diagnosis, endotracheal intubation, foreign body aspiration, general anesthesia, left main bronchus, recurrent pneumonia, rigid bronchoscopy, tooth aspiration

## Abstract

Foreign body aspiration (FBA) in adults is rare, and tooth aspiration represents an exceptionally uncommon subset. Even rarer is the prolonged retention of a tooth in the left main bronchus, which is anatomically less favorable for aspiration than the right. However, individual airway variations may predispose to such occurrences.

We report the case of a 56-year-old woman with an 11-year history of recurrent “pneumonia-like” episodes characterized by chronic productive cough, intermittent fever, hemoptysis, and pleuritic chest pain. She had been repeatedly treated with empirical antibiotic regimens - including azithromycin, amoxicillin-clavulanate, and levofloxacin - and intermittent corticosteroids, resulting in only transient symptomatic relief. Subsequently, she developed recurrent profuse watery diarrhea following prolonged antibiotic exposure, initially managed with oral metronidazole but complicated by recurrence and eventual toxic megacolon secondary to severe *Clostridioides difficile* colitis. She was referred to our tertiary care center for possible surgical intervention and was successfully managed conservatively with oral vancomycin and intravenous metronidazole. During hospitalization, further evaluation of her chronic respiratory symptoms with CT and bronchoscopy revealed a retained incisor lodged in the left main bronchus. Flexible bronchoscopy was unsuccessful because of dense impaction and bleeding; however, definitive extraction was achieved using rigid bronchoscopy under general anesthesia. Post-procedure recovery was uneventful, and six-month follow-up demonstrated complete clinical and radiologic resolution.

This case highlights the importance of considering FBA in adults with chronic, unexplained pulmonary symptoms, the potential consequences of repeated empirical antibiotic therapy - particularly in resource-limited settings - and the critical role of rigid bronchoscopy in definitive management.

## Introduction

Foreign body aspiration (FBA) in adults is uncommon, with an incidence ranging from 0.16% to 0.33% among respiratory complaints [1]. It often presents with nonspecific features that mimic chronic respiratory infections, asthma, or malignancy [2]. In contrast to children, adult presentations are frequently delayed due to low clinical suspicion and vague symptoms [3]. Prolonged retention of a bronchial foreign body may lead to recurrent respiratory infections, chronic inflammation, bronchiectasis, hemoptysis, or persistent airway obstruction.

Aspiration during airway manipulation, especially endotracheal intubation, represents a recognized yet underreported complication [4]. Dental trauma is a recognized anesthesia-related adverse event, with reported incidence ranging from 0.02% to as high as 12% among patients undergoing tracheal intubation [5], with tooth aspiration being a rare but serious sequela. Patients with poor dentition, advanced age, or difficult airways are at greater risk of peri-intubation dental injury and potential aspiration of fractured teeth.

Although the right main bronchus is more commonly involved due to its vertical alignment, the left main bronchus can occasionally serve as a site of impaction, particularly in the presence of anatomical variations [6]. Clinical presentations range from persistent cough and recurrent pneumonia to hemoptysis or airway obstruction. Imaging may suggest but not confirm the diagnosis, making bronchoscopy the definitive diagnostic and therapeutic tool. However, prolonged retention of an aspirated dental foreign body may remain unrecognized when symptoms are intermittent and nonspecific, highlighting the importance of maintaining clinical suspicion even in patients with a remote history of airway manipulation.

We present a rare case of delayed diagnosis of an aspirated incisor retained for 11 years in the left main bronchus following difficult intubation. The unusually prolonged retention period, dental origin of the foreign body, and its location in the left main bronchus make this case particularly noteworthy and highlight the diagnostic challenges associated with long-standing bronchial foreign bodies.

## Case presentation

A 56-year-old woman from a remote rural region was referred to our tertiary care center for evaluation and management of suspected toxic megacolon. She had initially presented to a local healthcare facility with profuse watery diarrhea, diffuse abdominal pain, fever, and progressive abdominal distension following multiple prolonged courses of empirical antibiotics prescribed for chronic respiratory symptoms. Over the preceding years, she had repeatedly received various antibiotic regimens - including azithromycin, amoxicillin-clavulanate, and levofloxacin - along with intermittent short courses of prednisolone, with only temporary symptomatic relief. The diarrhea had initially improved with oral metronidazole therapy but recurred twice over subsequent months. Abdominal radiography at the referring center demonstrated marked colonic dilatation consistent with toxic megacolon, most likely secondary to severe *Clostridioides difficile* colitis, prompting referral to our institution because of the potential need for urgent surgical intervention.

On arrival, she was managed conservatively with bowel rest, intravenous fluids, oral vancomycin, and intravenous metronidazole under close multidisciplinary supervision. Her gastrointestinal symptoms gradually improved, and surgical intervention was ultimately not required.

During hospitalization, a detailed review of her medical history revealed an 11-year history of recurrent lower respiratory tract infections characterized by persistent productive cough with mucoid sputum, intermittent low-grade fever, occasional hemoptysis, and left-sided pleuritic chest pain. Despite repeated treatment by local practitioners, her respiratory symptoms had recurred within weeks of each antibiotic course, with no definitive diagnosis established.

Her medical history was significant for type 2 diabetes mellitus, hypertension, hyperlipidemia, hypothyroidism, and heart failure with reduced ejection fraction. She denied any history of smoking, alcohol use, or occupational exposure to dust or chemicals. Surgical history included a cesarean section and a cholecystectomy performed 11 years earlier under general anesthesia. According to her family, she had experienced a gum infection following that surgery. No evaluation was undertaken at the time because she remained asymptomatic immediately postoperatively and had poor baseline dental hygiene (Figure 1).

**Figure 1:**
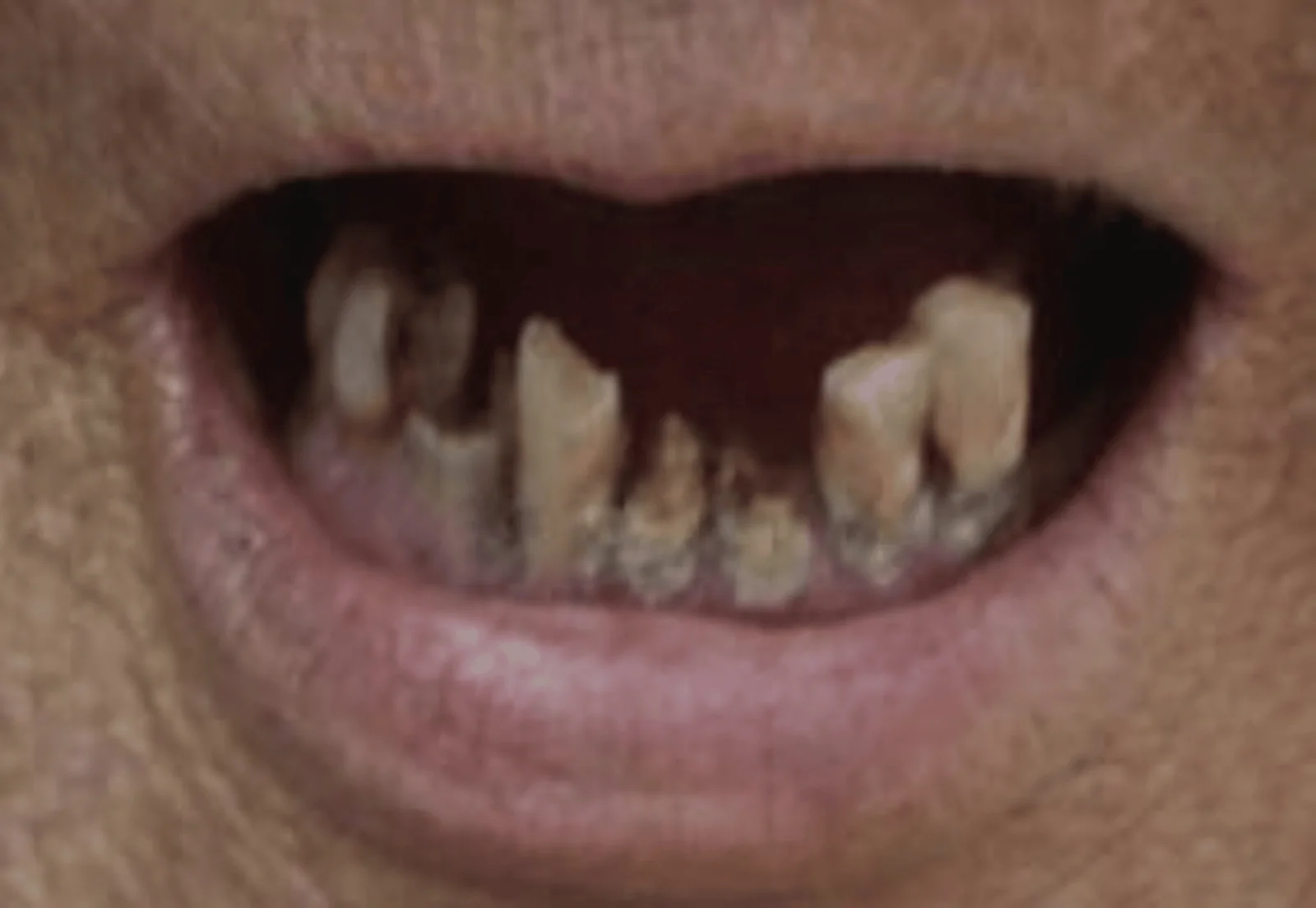
Severe dental caries and poor dentition, representing the likely source of the aspirated tooth

Following stabilization of her gastrointestinal condition, further evaluation of her chronic pulmonary symptoms was undertaken. On examination, the patient appeared stable and comfortable. Vital signs were within normal limits, with an oxygen saturation of 97% on room air. Chest auscultation revealed reduced breath sounds and coarse crackles over the left lower lung field. There were no signs of respiratory distress, clubbing, or cyanosis.

Routine laboratory investigations - including complete blood count, ESR, CRP, liver function tests, and renal function tests - were within normal limits (Table 1). Sputum examination was negative for acid-fast bacilli, and sputum cytology showed no malignant cells (Table 2).

**Table 1:** Laboratory investigations (quantitative)

Parameter	Value	Reference Range
Hemoglobin	12.8 g/dL	12-16 g/dL
WBC	10.2 ×10⁹/L	4-11 ×10⁹/L
Platelets	286 ×10⁹/L	150-450 ×10⁹/L
ESR	18 mm/hour	0-20 mm/hour
CRP	2.8 mg/L	<5 mg/L
Creatinine	0.8 mg/dL	0.5-1.0 mg/dL
Aspartate aminotransferase	28 U/L	10-40 U/L
Alanine aminotransferase	24 U/L	7-56 U/L
Bilirubin	0.6 mg/dL	0.2-1.2 mg/dL
International normalized ratio	1.1	0.8-1.2

**Table 2:** Sputum evaluation (qualitative) AFB - acid-fast bacilli; BAL - bronchoalveolar lavage

Parameter	Patient Value	Reference Range	Interpretation
BAL culture	Sterile	No growth	No active infection
Sputum AFB smear	Negative	Negative	No tuberculosis suspected
Cytology (sputum)	Negative	Negative	No malignant cells

Chest radiography demonstrated persistent left perihilar opacity with associated volume loss. High-resolution computed tomography of the chest revealed a hyperdense tooth-like foreign body lodged within the proximal left main bronchus, causing partial obstruction with distal segmental collapse, air trapping, and bronchiectatic changes suggestive of chronic airway obstruction (Figure 2). No pleural effusion or mediastinal shift was identified.

**Figure 2:**
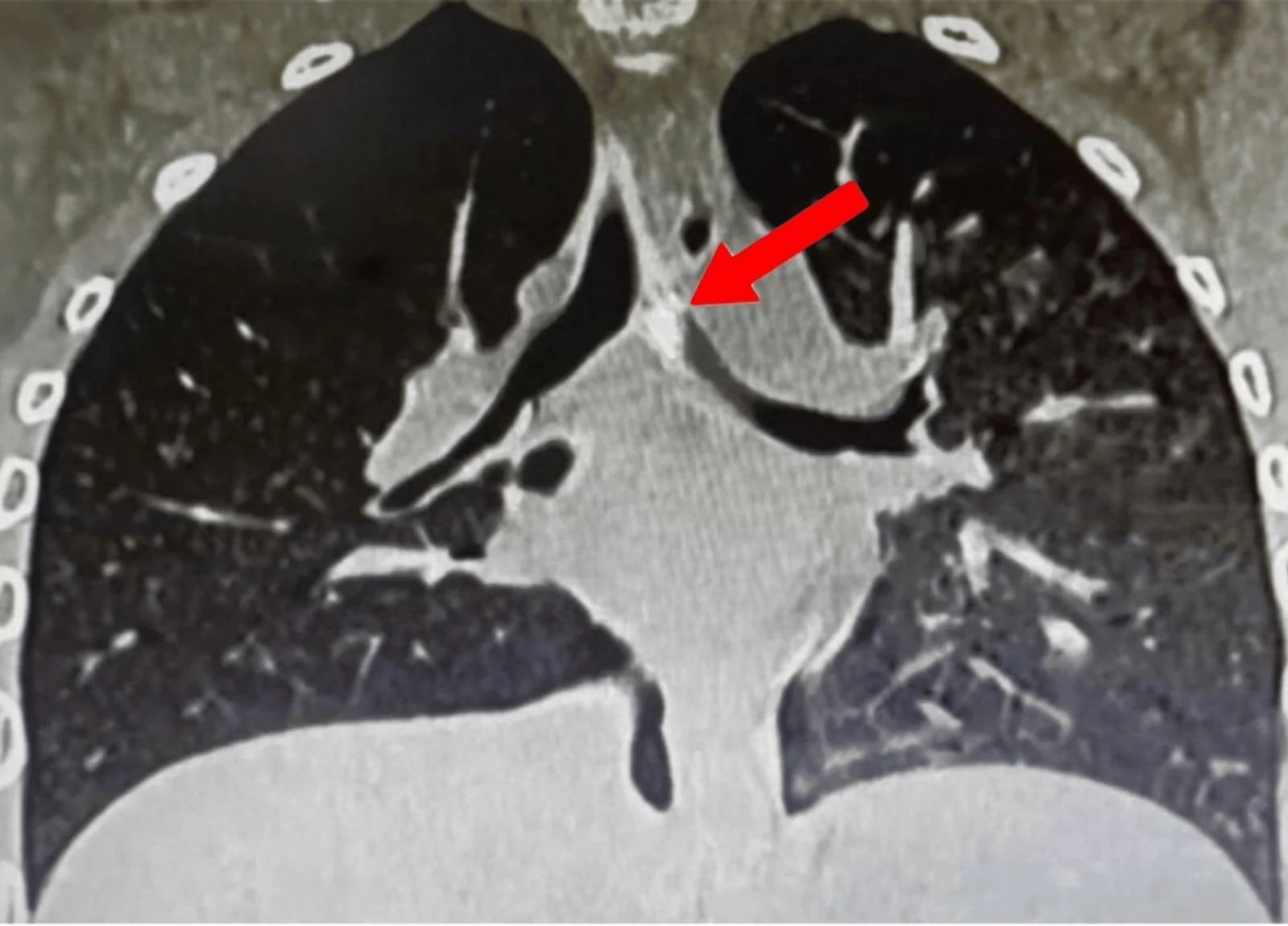
Coronal chest CT demonstrating a foreign body lodged within the left main bronchus (red arrow) with associated chronic airway changes

Given the chronicity of symptoms and characteristic radiologic findings, a diagnosis of retained bronchial foreign body was established, and flexible bronchoscopy was scheduled for diagnostic confirmation and possible extraction.

Flexible bronchoscopy demonstrated a hard, whitish, irregular foreign body nearly occluding the left main bronchus and surrounded by friable mucosa with extensive granulation tissue (Figure 3). Gentle attempts at dislodgement resulted in brisk bleeding that obscured visualization, necessitating termination of the procedure to avoid airway injury. The patient remained hemodynamically stable throughout.

**Figure 3:**
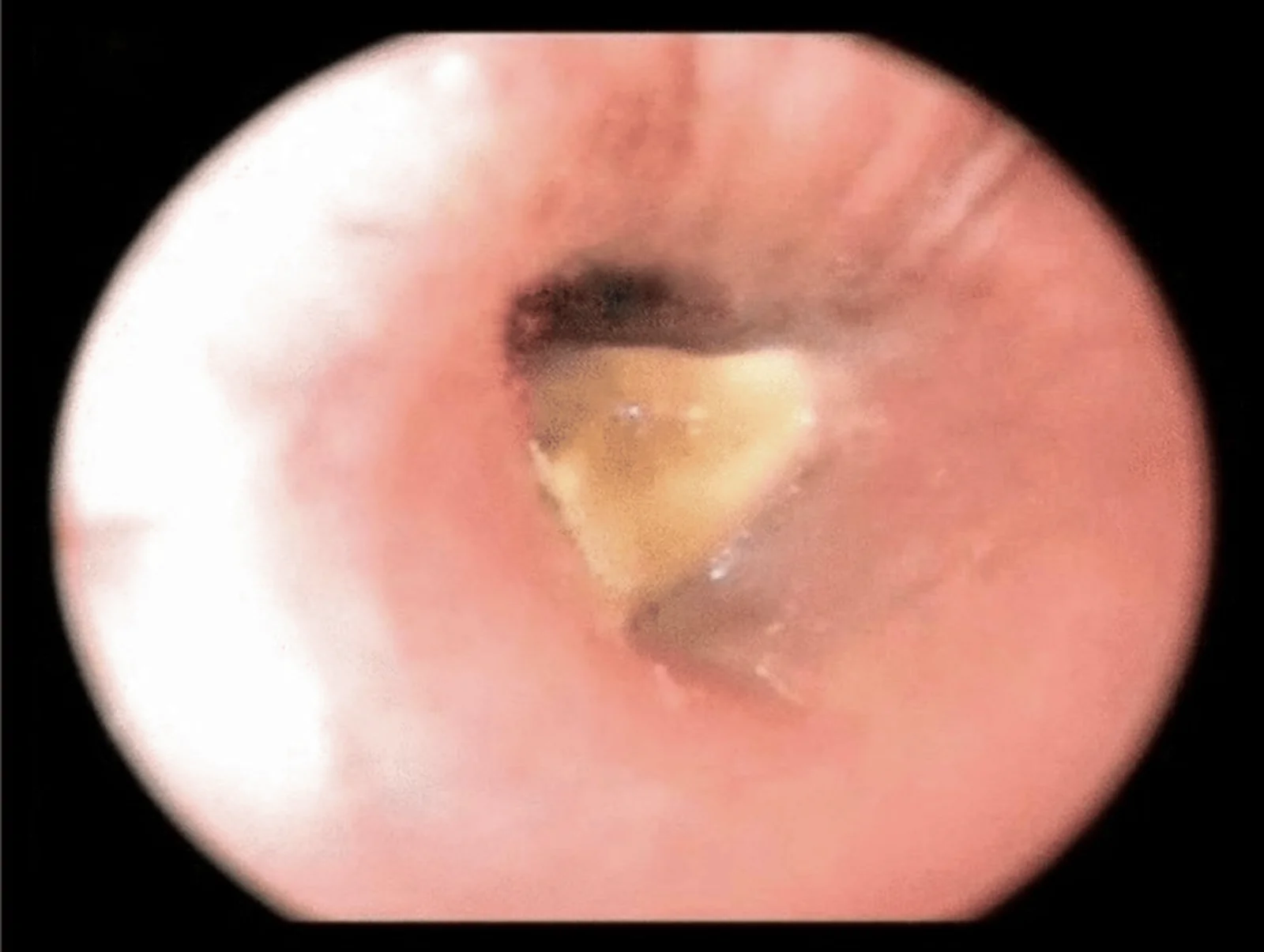
Bronchoscopic view showing an impacted tooth within the bronchial lumen surrounded by granulation tissue

Correlation with her prior surgical history and the missing tooth following cholecystectomy strongly suggested peri-intubation aspiration of the incisor (Figure 4). Definitive removal by rigid bronchoscopy under general anesthesia was therefore planned.

**Figure 4:**
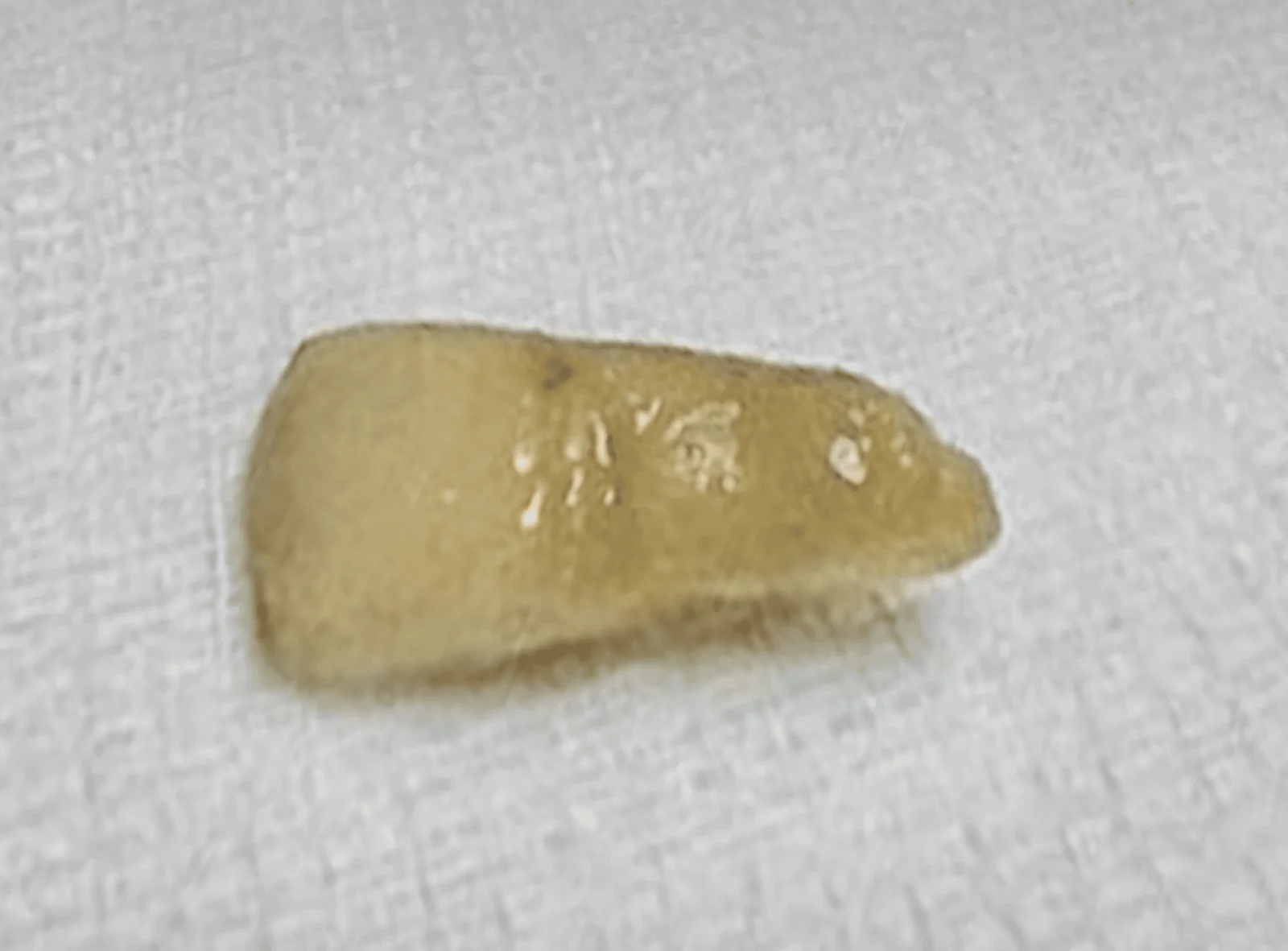
Extracted tooth following successful bronchoscopic removal

The following day, rigid bronchoscopy revealed a lower incisor tightly impacted within the left main bronchus. Using optical forceps, the tooth was carefully disengaged with gentle rotational maneuvers and extracted intact. Minor procedural bleeding was successfully controlled with topical 1:10,000 adrenaline. Post-extraction bronchoscopy confirmed restoration of bronchial patency with mild residual mucosal edema.

Postoperatively, the patient was monitored in the intensive care unit for 24 hours because of mild hypoxemia (SpO₂ 90-92%) and scant blood-stained secretions, both of which resolved spontaneously. Follow-up bronchoscopy performed 48 hours later demonstrated a clear bronchial lumen without residual obstruction.

The patient was discharged on the fifth postoperative day with complete resolution of respiratory symptoms. At one-month follow-up, chest radiography demonstrated full re-expansion of the left lung, and at six months she remained asymptomatic, with no clinical or radiologic evidence of recurrence or complications.

She was counseled regarding the importance of dental evaluation prior to future intubation procedures, maintenance of oral hygiene, and consideration of appropriate dental protection measures, including bite blocks in selected circumstances during airway manipulation.

## Discussion

FBA in adults is rare and often overlooked due to its subtle and variable presentation. Unlike pediatric cases - where aspiration events are often witnessed - adult cases are frequently missed until complications, such as chronic infection, collapse, or hemoptysis, appear [7].

In this patient, recurrent episodes of “pneumonia” confined to the same lung segment over several years raised suspicion for chronic localized obstruction. However, the absence of early imaging and the tendency for empirical antibiotic therapy in resource-limited settings delayed diagnosis. The radiological finding of a hyperdense object within the bronchus was pivotal in redirecting the diagnostic approach.

Aspiration of a tooth during intubation, although exceedingly rare, is a recognized complication. Difficult airway management, poor dentition, and reduced gag reflex under anesthesia are key predisposing factors [8]. In our case, the aspiration likely occurred during the difficult intubation 11 years earlier, with the tooth initially remaining asymptomatic until chronic inflammation and granulation tissue formation subsequently led to recurrent, intermittent infection-like symptoms.

Prolonged retention of a bronchial foreign body can lead to a spectrum of complications-ranging from localized bronchiectasis and recurrent pneumonitis to irreversible lung collapse and hemoptysis [9]. These complications are often mistaken for tuberculosis or malignancy, particularly in endemic regions.

Bronchoscopic management remains the cornerstone of diagnosis and therapy. Flexible bronchoscopy offers a minimally invasive approach for diagnosis and removal in most cases. However, hard, irregular objects, such as teeth or metallic fragments, often necessitate rigid bronchoscopy, which provides superior airway control, larger working channels, and better suction capabilities [10]. In our patient, flexible bronchoscopy established the diagnosis, but extraction attempts were limited by bleeding and poor visualization, making rigid bronchoscopy the definitive intervention.

This case also underscores a broader public health concern: the overuse of antibiotics without definitive diagnosis, especially in peripheral and resource-poor regions. Repeated empirical treatments not only delay identification of the actual cause but also contribute to antimicrobial resistance, *Clostridioides difficile* infections, and unnecessary healthcare expenditure.

Finally, this case reinforces the value of simple historical clues - in this instance, the loss of a tooth post-intubation - that can unlock a diagnostic mystery spanning years. It serves as a reminder for anesthesiologists and pulmonologists alike to consider dental inspection both before and after airway manipulation, and for clinicians to maintain a high index of suspicion for FBA in adults with unexplained or refractory pulmonary findings.

## Conclusions

This case illustrates an unusual but clinically significant complication of intubation: tooth aspiration. In patients with poor dentition, persistent or localized pulmonary symptoms following surgery or intubation should prompt consideration of FBA. Early imaging and bronchoscopy are crucial for diagnosis, while rigid bronchoscopy remains the definitive therapeutic tool when flexible methods fail. Preventive dental assessment and careful airway management can avert such overlooked yet preventable complications.
